# Interactions between macro-nutrients’ intake, FTO and IRX3 gene expression, and FTO genotype in obese and overweight male adolescents

**DOI:** 10.1080/21623945.2019.1693745

**Published:** 2019-11-27

**Authors:** Saeid Doaei, Naser Kalantari, Pantea Izadi, Tuire Salonurmi, Alireza Mosavi Jarrahi, Shahram Rafieifar, Ghasem Azizi Tabesh, Ghazaleh Rahimzadeh, Maryam Gholamalizadeh, Mark O. Goodarzi

**Affiliations:** a Research Center of Health and Environment, Guilan University of Medical Sciences, Rasht, Iran; b Student Research Committee, Cancer Research Center, Shahid Beheshti University of Medical Sciences, Tehran, Iran; c Department of Community Nutrition, School of Nutrition and Food Sciences, Shahid Beheshti University of Medical Sciences, Tehran, Iran; d Department of Medical Genetics, School of Medicine, Tehran University of Medical Sciences, Tehran, Iran; e Department of Internal Medicine, Oulu University Hospital and University of Oulu, Oulu, Finland; f Faculty of Medical School, Shahid Beheshti University of Medical Sciences, Tehran, Iran; g Health Promotion and Education Department, Ministry of Health, Tehran, Iran; h Department of Medical Genetics, School of Medicine, Shahid Beheshti University of Medical Sciences, Tehran, Iran; i Institute for Intelligent Systems Research and Innovation (IISRI), Deakin University, Geelong Waurn Ponds, Australia; j Division of Endocrinology, Diabetes and Metabolism, Department of Medicine, Cedars-Sinai Medical Center, Los Angeles, CA, USA

**Keywords:** FTO, IRX3, macronutrients, genotype, carbohydrate, protein, fat

## Abstract

This study is the first to identify the effects of FTO genotype on the interactions between the level of macro-nutrients intake and the expression level of fat mass and obesity associated (FTO) and homeobox transcription factor iriquois-3 (IRX3) genes This longitudinal study was carried out on 84 overweight and obese adolescent boys in Tehran, Iran. The rs9930506 SNP in *FTO* was genotyped at baseline and the level of *FTO* and *IRX3* expression in PBMCs and macro-nutrients’ intake were assessed at baseline and after 18 weeks of the intervention. The results identified that the higher carbohydrates intake significantly up-regulated the FTO gene (P = 0.001) and down-regulated the IRX3 gene (P = 0.01). Protein intake up-regulated the FTO gene (P = 0.001). In carriers of GG genotype of FTO gene, the amount of dietary carbohydrate had a positive association with FTO gene expression (p = 0.001, and p = 0.04, respectively). In AA/AG carriers, dietary protein was positively associated with FTO gene expression (p = 0.001) and dietary carbohydrate was negatively associated with IRX3 gene expression (P = 0.04). Therefore, dietary carbohydrateseems to be associated with FTO and IRX3 genes expression. These associations are influenced by FTO genotype.

## Introduction

Obesity in childhood, especially during the second decade of life, has been proposed as a strong predictor of adult obesity [1,2] and is associated with many chronic diseases including diabetes, cardiovascular disease, cancer, psychological conditions [3]. It is a multifactorial disorder caused by both environmental and genetic factors [4,5] and recent studies have reported that genes contribute to 25% to 40% of childhood obesity [5].

Some genes such as the fat mass and obesity-associated (FTO) gene are reported to be strongly associated with obesity and overweight [4,5] after the age of 7 y [6]. The FTO gene, located in the chromosome region 16q12.2, is expressed in many tissues, especially in brain and hypothalamus [4]. It is indicated that FTO genotype had a strong association with body composition and body weight. FTO gene has been supposed to have an important role in food intake regulation and basal metabolic rate [7]. Results from recent studies identified that there is a mutual interaction between FTO gene and the intake of dietary macronutrients [8,9]. Some studies indicated that dietary macronutrients including fats, carbohydrates and proteins might affect FTO gene expression [8]. On the other hand, the effects of dietary macronutrients on body weight, hormone secretion, and appetite are influenced by FTO genotype [9,10].

Recent studies have tried to explore the interaction of macronutrient composition with FTO gene expression [11,12]. Dietary composition especially dietary carbohydrates, as the main source of calorie in a regular diet, have an important role in regulation energy balance and body weight [12]. Also, the level of protein intake could modify the association between FTO genotype and BMI [13]. Moreover, recent studies reported that some of the effects of the FTO gene on body weight may be due to the effects of FTO gene on the expression level of Iroquois-related homeobox 3 (IRX3) [14]. The IRX3 gene is a member of Iroquois homeobox gene family, known to play multiple roles in the early steps of neural development. It is reported that the IRX3 gene expression is controlled by sequence of intron 1 in the FTO gene [15]. The expression level of this gene in hypothalamus is associated with calorie intake and body composition [16].

In order to understand whether the intake of macronutrients is associated with FTO and IRX3 genes, we conducted a field trial study to investigate the interactions between changes in the amount of dietary carbohydrate, protein and fat, with the expression level of FTO and IRX3 genes and FTO genotype in obese and overweight male adolescents.

## Methodology

This study was an ancillary study within a randomized, controlled, school-based trial that carried out a comprehensive weight-reduction program. The original study had an intervention group (n = 44) and a control group (n = 40). Data from the control and intervention groups were combined for purposes of the present study (n = 84). A personalized diet was implemented for each participant. In addition, parents were provided an educational session on healthy diet to creating a supportive environment at home. The study involved students in two high schools (7th, 8th, and 9th grade students) of a district of Tehran city that was chosen randomly (district 5).

### Research context and subject recruitment

The following details are presented in accordance with the CONSORT reporting guidelines for randomized trials of non-pharmacologic treatment (sup. 1). This study was a field trial and details of the trial have been published elsewhere [17]. In brief, participants were adolescent boys, overweight or obese (BMI ≥ +1 z-scores), aged 12–16 years. The specific exclusion criteria included: uncontrolled chronic diseases affecting weight such as thyroid dysfunction or psychiatric diseases and taking medications affecting weight. Five hundred and forty students in two boys’ high schools (including grades 7–9) of a randomly chosen district of Tehran city (District 5) attended an information session and 246 were eligible to participate in the parent trial. Of these, 96 expressed interest in participating in the ancillary study, 84 enrolled and consented to the blood sampling at baseline and week 18, and 62 provided both baseline and week 18 blood samples. Thus, 62 participants were included in the analysis. The inclusion criteria were age between 12 and 16 years and BMI percentile reported as ⩾ 85th percentile for age and sex. Non-inclusion criteria included diagnosed weight-related diseases (including hypothyroidism, insulin resistance, and Cushing’s syndrome) and use of weight-related medications (including drugs for diabetes, such as insulin, thiazolidinediones, and sulfonylureas; antipsychotic drugs such as haloperidol, clozapine, and lithium; and antidepressant drugs like amitriptyline, paroxetine, and sertraline) determined by self-report by the participants. All measures were taken between morning and noon at baseline and after 18 weeks of intervention.

### Anthropometric measures

The measures of baseline and week 18 were obtained by trained personnel. The height of students was measured with a calibrated tape line fastened to a wall. A bio-impedance analysis scale (Omron BF511 Kyoto, Japan) was used to measure weight, BMI, %body fat, and %skeletal muscle after entering their age, gender, and height. This device is a digital, mobile, and non-invasive device that has eight electrodes that sends an extremely weak electrical current of 50 kHz and less than 500 πA through the body to determine the amount of fat tissue. The validity of this device has been confirmed in a previous study [18]. All data were classified according to the z-score guidelines defined by WHO recommendations [19] and recently published papers (for %body fat and %skeletal muscle [20]).

### Quantitative real-time PCR

At baseline and week 18, blood samples (5 ml) were collected of all students who participated in the study, transferred to EDTA tubes and stored at −80°C. Total RNA from peripheral blood mononuclear cells (PBMCs) was subsequently isolated using the GeneAll RNA extraction kit (GeneAll, South Korea), cDNA synthesis was performed using the GeneAll cDNA synthesis kit (GeneAll, South Korea), and mRNA expression levels were determined using the Opticon real-time PCR detection system (Bio-Rad Laboratories, California). Reactions were carried out in duplicate using SYBR Green Gene Expression Master Mix (Cat. no. 638317; Takara, Japan). The HPRT gene was used as the reference gene for normalization, chosen because of its stable expression in blood cells. Quantification of transcripts of interest relative to the internal housekeeping control gene HPRT was performed using the 2^−ΔΔCt^ method and expressed as fold change. Changing *FTO* and *IRX3* expression was evaluated using the REST (Relative Expression Software Tool) software. Data on changes of gene expression were transferred to SPSS software in order to analyse their relationships with dietary intake and *FTO* genotype.

### Genotyping

The DNA extraction kit manufactured by GeneAll was used to extract and purify DNA samples. The NanoDrop device (Thermo Scientific, Wilmington, DE, USA) was used to quantify DNA concentration. The optical density (OD) of the samples was measured at a wavelength of 260–280 nm. Moreover, to check the quality of the extracted DNA, electrophoresis using the agarose gel technique was used. In brief, genomic DNA was amplified by PCR using the Taq DNA Pol 2X Master Mix Red (cat. No A180301; Ampliqon, Denmark). The PCR products were sent to GeneAll for DNA sequencing. The rs9930506 SNP in *FTO* was genotyped in all subjects and the quality and average length of the sequence library for each sample was assessed using the Chromas software (version 2.33, http://www. technelysium.com.au/chromas.html).

### Macronutrients’ intake

Usual Macronutrients’ intakes of participants were examined by a validated 168-item semi-quantitative FFQ [10]. The FFQ consisted of 168 food items with standard portion sizes commonly consumed by Iranian people. A trained interviewer administered the FFQ through face-to-face interviews. Macronutrients’ consumption frequencies were converted to grams per day by using household measures. Daily intakes of energy were measured for each person by using the modified US Department of Agriculture food consumption database, which was modified for Iranian foods.

### Statistical analysis

We expected that changes in macronutrients’ intake in the subjects are significantly associated with the level of genes expression over a period of 18 weeks. We aimed to test the hypothesis that there is an association between changes of the level of FTO and IRX3 genes expression with macronutrients’ intake over a time period regardless of the effect of physical activity. All associations were evaluated under dominant models. We compared pre- and post-intervention values using the REST software and analysed means of two groups using independent t-test. Multiple linear regression was used to determine the relationship between changes in FTO gene expression with changes of macronutrients’ intake of subjects with different FTO genotypes after adjusting for age, physical activity, and calorie intake. We confirmed that the assumptions of the linear regression model were met. Statistical analyses were performed using SPSS version 23.0 (IBM SPSS Statistics for Windows, IBM Corp., Armonk, NY, USA). The results were considered statistically significant at P < 0.05.

### Ethics approval and consent to participate

This study was approved by the Ethics Committee of Shahid Beheshti University of Medical Sciences (Reference Number: Ir.sbmu.nnftri.rec. 1394.22), Tehran, Iran. The schools that were involved in this study were asked permission to be part of this trial and consented for their students to participate. The details of the study were explained to students and their parents with an explanatory letter and written informed consent was obtained from both parents and students prior to joining the project.

## Results

All measurement data were normally distributed (P > 0.05). At baseline, the obese participants had higher BMI, %body fat, and lower %skeletal muscle compared to overweight subjects (p = 0.00) (Table 1). No significant differences were found between two groups in dietary intake, physical activity, and FTO and IRX3 gene expression at baseline.

**Table 1.** Characteristics of study participants in obese and overweight adolescent boys at baseline.

	OverWeight (n = 30)	Obese (n = 32)	P
Age(year)	13.89 ± 0.914	13.90 ± .918	0.67
BMI(Kg/m^2^)	23.5731 ± 1.62	29.1592 ± 2.94498	0.00
FM%	24.1214 ± 5.66	31.1673 ± 5.02230	0.00
SM%	36.4715 ± 2.39	33.6735 ± 2.09352	0.00
PA(MET-minute per week)	1744 ± 2355.46	1894 ± 2598.27318	0.31
Calorie(Kcal)	2168 ± 772.35	2192 ± 1134.02343	0.92
Protein intake(gr/day)	166.12 ± 280.18	88.29 ± 48.95	0.16
Carbohydrate intake(gr/day)	220.41 ± 86.16	248.49 ± 105.12	0.26
Fat intake(gr/day)	177.33 ± 89.89	170.36 ± 75.43	0.74
FTO	3.1764 ± 10.63104	1.7829 ± 2.03810	0.5
IRX3	11.2229 ± 4.57277	2.8662 ± 3.92054	0.38

The higher carbohydrates intake significantly up-regulated the FTO gene (P = 0.001) and down-regulated the IRX3 gene (P = 0.01). Protein intake up-regulated the FTO gene (P = 0.000), with no significant effect on IRX3 expression (P = 0.57). There was no significant association between fat intake and IRX3 and FTO expression (P = 0.65, P = 0.26, respectively). (Table 2)

**Table 2.** The association of changes of macronutrients’ intake with changes in FTO and IRX3 gene expression over a period of 18 weeks.

	FTO		IRX3	
	Beta	P	Beta	P
Protein intake(gr/day)	0.65	0.000	−0.07	0.57
Carbohydrate intake(gr/day)	0.97	0.001	−1.07	0.01
Fat intake(gr/day)	−0.05	0.65	−1.16	0.26

Adjusted for age, Kcal, BMI, FM, SM, and PA.

Considering FTO genotype, in carriers of GG genotype of FTO gene, the amount of dietary protein and carbohydrate had significant associations with FTO gene expression (p = 0.001, and p = 0.04, respectively). In AA/AG carriers, dietary carbohydrate was significantly associated only with IRX3 gene expression (P = 0.04). (Table 3)

**Table 3.** The association of macronutrients’ intake with FTO and IRX3 gene expression based on different FTO genotypes.

	AA/AG				GG			
	FTO		IRX3		FTO		IRX3	
	Beta	P	Beta	P	Beta	P	Beta	P
Protein intake	0.66	0.000	−0.09	0.57	0.03	0.94	−0.01	0.98
Carbohydrate intake	0.49	0.21	−0.74	0.04	1.56	0.04	−0.7	0.43
Fat intake	−0.01	0.99	−0.19	0.49	−0.57	0.07	−0.67	0.29

Adjusted for age, Kcal, BMI, Fat mass, Skeletal Muscle, and Physical Activity.

## Discussion

This study is the first to identify the concurrent interactions of FTO genotype, FTO and IRX3 expression, and dietary macronutrients. The results indicate that increased amount of protein and carbohydrates intake significantly up-regulated the FTO gene. Increased carbohydrates intake also down-regulated the IRX3 gene. In AA/AG carriers, dietary protein was positively associated with FTO expression and carbohydrate was negatively associated with IRX3 gene expression. In carriers of GG genotype of FTO gene, the amount of dietary carbohydrate had a positive association only with FTO gene expression.

However, that the results of recent studies on the effect of dietary macronutrients on FTO and IRX3 gene expression were inconsistent. In regards to dietary carbohydrate, Poritsano et al. reported that higher carbohydrate especially glucose intake up-regulated FTO gene expression [21]. On the other hand, Boender et al. found that increased sucrose administration reduced FTO gene expression [22]. Also, Olszewski et al. found no changes in hypothalamic FTO expression after a 48-h palatable sucrose feeding in mice. In regards to dietary protein, Olszewski et al. found that anorexigenic Leucine had reduced FTO gene expression in organotypic cultures of the hypothalamus at 48 h post intervention [23]. While Johansson et al. found that Leucine intake had increased FTO gene expression at 48 h post intervention [24]. The exact mechanism of the changes of FTO gene expressions in response to the different type of dietary macronutrients remained unclear. FTO may play a key role in the relationship between amino acid levels and MTORC1 function [25]. Cheung et al. reported that FTO gene expression is downregulated by essential amino-acid deprivation [26]. The FTO gene is involved in amino acids pairing with MTORC1 signalling and in the absence of FTO, MTORC1 signalling is impaired [26].

Our study found no association between fat intake and gene expression. Zhong et al. found that a high-fat diet in comparison with a normal carbohydrate diet in mice did not change FTO gene expression [27,28]. Inconsistent with this study, Nowacka-Woszuk et al. reported that a high-fat diet could lead to an increase FTO and IRX3 genes expression in white adipose cells [29]. Ronkainen et al. investigated the relationship between dietary fat, the FTO expression and the IRX3 expression in rats. The high-fat diet could influence on the level of the IRX3 expression and suppression of FTO increases IRX3 expression [30].

No study was found on the concurrent interactions of FTO genotype, FTO and IRX3 expression, and dietary macronutrients. Few studies are carried out on the effect of dietary macronutrients on the associations between the FTO genotype and FTO and IRX3 genes expression. Sonestedt et al. reported that the association between FTO genotype and obesity is restricted to the groups of individuals with high-fat and low-carbohydrate diets [13]. Interestingly, another study confirmed that the FTO rs9939609 polymorphism was not associated with BMI in adult with higher carbohydrate intakes [31]. Tanaka et al. found that the FTO polymorphism (rs1421085) was associated with BMI in the group with higher protein intake [27]. The present study was the first that indicated the effect of FTO genotype on the association between dietary macronutrients and FTO and IRX3 genes expression and found that higher protein intake can up-regulate FTO gene expression in carriers of AA/AG genotype, while higher carbohydrate intake can up-regulate FTO gene expression in GG genotype. Moreover, higher carbohydrate intake can down-regulate IRX3 expression in AA/AG genotypes, NOT in GG genotype.

## Conclusion

FTO gene polymorphism is associated with dietary composition. The intake level of protein and carbohydrates significantly up-regulated the FTO gene and carbohydrates intake down-regulated the IRX3 gene. These findings highlighted the importance of FTO gene sequence on the possible link between dietary intake and gene expression and related functions. However, our study had some limitations including Small sample size, short period of follow-up, and genotyping for only one polymorphism. Further studies are needed to increase our understanding of the association between FTO and IRX3 gene and dietary intake.

## References

[1] WHO Obesity: preventing and managing the global epidemic. WorldHealth Organization; 2000 (Technical Report Series consultation). 11234459

[2] Strauss R, Pollack HA Epidemic increase in childhood overweight, 1986-1998. JAM. 2006;A:22,101–109. 10.1001/jama.286.22.2845 11735760

[3] Hubbard VS Defining overweight and obesity: what are the issues? Am J Clin Nutr. 2000;72:1067–1068. 11063427 10.1093/ajcn/72.5.1067

[4] Spadafora R The key role of epigenetics in human disease. N Engl J Med. 2018;379(4):400. 10.1056/NEJMc1805989 30048067

[5] Herrera BM, Keildson S, Lindgren CM Genetics and epigenetics of obesity. Maturitas. 2011;69(1):41–49. 21466928 10.1016/j.maturitas.2011.02.018 PMC3213306

[6] Hakanen M, Raitakari OT, Lehtimäki T, et al. FTO genotype is associated with body mass index after the age of seven years but not with energy intake or leisure-time physical activity. J Clin Endocrinol Metab. 2009;94:1281–1287. 19158205 10.1210/jc.2008-1199

[7] Kalantari N, Mohammadi NK, Izadi P, et al. A haplotype of three SNPs in FTO had a strong association with body composition and BMI in Iranian male adolescents. PLoS ONE. 2018;13(4):e0195589. 29677190 10.1371/journal.pone.0195589 PMC5909891

[8] Fischer J, Koch L, Emmerling C, et al. Inactivation of the Fto gene protects from obesity. Nature. 2009;458(7240):894–898. 19234441 10.1038/nature07848

[9] Rostami H, Samadi M, Yuzbashian E, et al. Habitual dietary. intake of fatty acids are associated with leptin gene expression in subcutaneous and visceral adipose tissue of patients without diabetes. Prostaglandins Leukot Essent Fatty Acids. 2017;126:49–54. 29031395 10.1016/j.plefa.2017.09.010

[10] Esfahani FH, Asghari G, Mirmiran P, et al. Reproducibility and relative validity of food group intake in a food frequency questionnaire developed for the Tehran lipid and glucose study. J Epidemiol. 2010;20(2):150–158. 20154450 10.2188/jea.JE20090083 PMC3900814

[11] Blundell JE, Lawton CL, Cotton JR, et al. Control of human appetite: implications for the intake of dietary fat. Annu Rev Nutr. 1996;16:285–319. 8839929 10.1146/annurev.nu.16.070196.001441

[12] Ludwig DS The glycemic index: physiological mechanisms relating to obesity, diabetes, and cardiovascular disease. JAMA. 2002;287(18):2414–2423. 11988062 10.1001/jama.287.18.2414

[13] Sonestedt E, Roos C, Gullberg B, et al. Fat and carbohydrate intake modify the association between genetic variation in the FTO genotype and obesity. Am J Clin Nutr. 2009 9 2;90(5):1418–1425. 19726594 10.3945/ajcn.2009.27958

[14] Lewis MT, Ross S, Strickland PA, et al. Regulated expression patterns of IRX-2, an Iroquois-class homeobox gene, in the human breast. Cell Tissue Res. 1999;296(3):549–554. 10370142 10.1007/s004410051316

[15] Smemo S, Tena JJ, Kim KH, et al. Obesity-associated variants within FTO form long-range functional connections with IRX3. Nature. 2014;507(7492):371e375. 24646999 10.1038/nature13138 PMC4113484

[16] Gholamalizadeh M, Doaei S, Akbari ME, et al. Influence of fat mass-and obesity-associated genotype, body mass index, and dietary intake on effects of iroquois-related homeobox 3 gene on body weight. Chin Med J (Engl). 2018;131(17):2112–2113. 30127222 10.4103/0366-6999.239309 PMC6111688

[17] Kalantari N, Mohammadi NK, Rafieifar S, et al. Indicator for success of obesity reduction programs in adolescents: body composition or body mass index? Evaluating a school-based health promotion project after 12 weeks of intervention. Int J Prev Med. 2017;8:73. 29026505 10.4103/ijpvm.IJPVM_306_16 PMC5634063

[18] Bosy-Westphal A, Later W, Hitze B, et al. Accuracy of bioelectrical impedance consumer devices for measurement of body composition in comparison to whole body magnetic resonance imaging and dual X-ray absorptiometry. Obes Facts. 2008;1(6):319–324. 20054195 10.1159/000176061 PMC6452160

[19] Cole TJ, Flegal KM, Nicholls D, et al. Body mass index cut offs to define thinness in children and adolescents: international survey. BMJ. 2007 7 26;335(7612):194. 17591624 10.1136/bmj.39238.399444.55 PMC1934447

[20] McCarthy HD, Samani‐Radia D, Jebb SA, et al. Skeletal muscle mass reference curves for children and adolescents. Pediatr Obes. 2014 8;9(4):249–259. 23776133 10.1111/j.2047-6310.2013.00168.x

[21] Poritsanos NJ, Lew PS, Fischer J, et al. Impaired hypothalamic Fto expression in response to fasting and glucose in obese mice. Nutr Diabetes. 2011;1:e19. 23154321 10.1038/nutd.2011.15 PMC3302139

[22] Boender AJ, van Rozen AJ, Adan RA Nutritional state affects the expression of the obesity-associated genes Etv5, Faim2, Fto, and Negr1. Obesity (Silver Spring). 2012;20(December(12)):2420–2425. 22627920 10.1038/oby.2012.128

[23] Olszewski PK, Fredriksson R, Olszewska AM, et al. Hypothalamic FTO is associated with the regulation of energy intake not feeding reward. BMC Neurosci. 2009;10(October(1)):12925. 10.1186/1471-2202-10-129 PMC2774323 19860904

[24] Johansson A Leucine intake affects brain activity and central expression of genes associated with food intake, energy homeostasis and reward [Student Thesis]. Mälardalen University, School of Sustainable Development of Society and Technology; 2011 Available from [Google Scholar]: http://mdh.diva-portal.org/smash/get/diva2:447251/FULLTEXT01.pdf

[25] Gulati P, Cheung MK, Antrobus R, et al. Role for the obesity-related FTO gene in the cellular sensing of amino acids. Proc Natl Acad Sci U S A. 2013 2 12;110(7):2557. 23359686 10.1073/pnas.1222796110 PMC3574930

[26] Cheung MK, Gulati P, O’Rahilly S, et al. FTO expression is regulated by availability of essential amino acids. Int J Obes (Lond). 2013 5;37(5):744–747. 22614055 10.1038/ijo.2012.77

[27] Tanaka T, Ngwa JS, Van Rooij FJ, et al. Genome-wide meta-analysis of observational studies shows common genetic variants associated with macronutrient intake. Am J Clin Nutr. 2013 5 1;97(6):1395–1402. 23636237 10.3945/ajcn.112.052183 PMC3652928

[28] Zhong T, Duan XY, Zhang H, et al. Angelica sinensis suppresses body weight gain and alters expression of the FTO gene in high-fat-diet induced obese mice. BioMed Res Int. 2017;2017:6280972. 29098158 10.1155/2017/6280972 PMC5632476

[29] Nowacka-Woszuk J, Pruszynska-Oszmalek E, Szydlowski M, et al. Nutrition modulates Fto and Irx3 gene transcript levels, but does not alter their DNA methylation profiles in rat white adipose tissues. Gene. 2017;610:44–48. 28179100 10.1016/j.gene.2017.02.002

[30] Ronkainen J, Huusko TJ, Soininen R, et al. Fat mass- and obesity-associated gene Fto affects the dietary response in mouse white adipose tissue. Sci Rep. 2015 3;18(5):9233. 10.1038/srep09233 PMC4363842 25782772

[31] Church C, Moir L, McMurray F, et al. Overexpression of Fto leads to increased food intake and results in obesity. Nat Genet. 2010;42(12):1086–1092. 21076408 10.1038/ng.713 PMC3018646

